# What factors preventing the older adults in China from living longer: a machine learning study

**DOI:** 10.1186/s12877-024-05214-8

**Published:** 2024-07-22

**Authors:** Shiyin Xiao, Yajie Bi, Wei Chen

**Affiliations:** 1 School of Psychology, Guizhou Normal University, Guiyang, China; 2 Center for Big Data Research in Psychology, Guizhou Normal University, Guiyang, China

**Keywords:** Machine learning, Older adults, Longevity, ADL, IADL, PDI

## Abstract

**Background:**

The fact that most older people do not live long means that they do not have more time to pursue self-actualization and contribute value to society. Although there are many studies on the longevity of the elderly, the limitations of traditional statistics lack the good ability to study together the important influencing factors and build a simple and effective prediction model.

**Methods:**

Based on the the data of Chinese Longitudinal Healthy Longevity Survey (CLHLS), 2008–2018 cohort and 2014–2018 cohort were selected and 16 features were filtered and integrated. Five machine learning algorithms, Elastic-Net Regression (ENR), Decision Tree (DT), Random Forest (RF), K-Nearest Neighbor (KNN), and eXtreme Gradient Boosting (XGBoost), were used to develop models and assessed by internal validation with CLHLS 2008–2018 cohort and temporal validation with CLHLS 2014–2018 cohort. Besides, the best performing model was explained and according to the variable importance results, simpler models would be developed.

**Results:**

The results showed that the model developed by XGBoost algorithm had the best performance with AUC of 0.788 in internal validation and 0.806 in temporal validation. Instrumental activity of daily living (IADL), leisure activity, marital status, sex, activity of daily living (ADL), cognitive function, overall plant-based diet index (PDI) and psychological resilience, 8 features were more important in the model. Finally, with these 8 features simpler models were developed, it was found that the model performance did not decrease in both internal and temporal validation.

**Conclusions:**

The study indicated that the importance of these 8 factors for predicting the death of elderly people in China and built a simple machine learning model with good predictive performance. It can inspire future key research directions to promote longevity of the elderly, as well as in practical life to make the elderly healthy longevity, or timely end-of-life care for the elderly, and can use predictive model to aid decision-making.

**Supplementary Information:**

The online version contains supplementary material available at 10.1186/s12877-024-05214-8.

## Introduction

Longevity, defined as the length of life, is a concept often associated with living to 90 or 100 years and is a goal deeply rooted in human aspirations across cultures and eras [1, 2]. In Chinese society, a common wish for the older adults is to ‘live to be a hundred.’ However, the current reality shows that many individuals face premature death after reaching old age, with the global life expectancy in 2019 being only 73.4 years [3]. This early mortality not only deprives the older adults of the chance to enjoy life and pursue self-fulfillment but also results in a loss of valuable knowledge and experience for society.

China, which houses one-fifth of the world’s older adults population [4], is particularly concerned with this demographic shift. The proportion of the population aged 65 and above was 10.5% in 2015 and is projected to rise to 26.1% by 2050 [5]. This trend is accompanied by a death rate that significantly exceeds the birth rate, as evidenced by the 9.02 million births and 11.1 million deaths in China in 2023, leading to a decrease in the total population by 2.08 million compared to the previous year [6]. Similar patterns are observed in other countries like Japan and South Korea [7], highlighting China and a global concern for aging populations and their health [8, 9]. Studying the factors that contribute to the death of older adults can provide critical insights into the key elements necessary for maintaining their health and promoting longevity. Our research, which focuses on the mortality patterns of China’s older adults, aims to offer valuable perspectives that can inform health policies and interventions not only in China but also in other countries grappling with similar demographic challenges.

Extensive research has been conducted on the factors influencing older adults mortality in China. For instance, Zeng and Shen [10] examined the link between psychological resilience and longevity among the older adults, discovering that resilience positively impacts the longevity of seniors aged 65 and above, with the effect intensifying at more advanced ages. Li et al. [11] investigated the connection between cognitive function and all-cause mortality in the older adults, revealing that moderate to severe cognitive impairments elevate the risk of death in this demographic. Fan and He [12] explored the association between self-rated health and all-cause mortality among the older adults, noting that those with poorer self-rated health faced a higher mortality risk compared to those with better self-perceived health. Zhang and Feldman [13] studied the decline in daily living abilities as a precursor to death in the older adults. Chen et al. [14] analyzed the impact of the overall Plant-Diet Index, a measure of plant-based diet intake, on older adults mortality and found that a higher PDI was associated with reduced all-cause mortality. Song et al. [15] researched the correlation between self-reported life satisfaction and life expectancy in the older adults, determining that increased life satisfaction correlates with a lower death rate and extended life expectancy.

These studies have identified that factors such as psychological resilience, cognitive function, self-rated health, ADLs, and the PDI significantly influence older adults mortality. However, they typically focus on one or a few variables at a time, which reflects a limitation inherent to traditional statistical analyses. A comprehensive examination of these factors could yield more insightful findings, such as identifying the most critical factors among them, assessing their predictive power regarding older adults mortality, and understanding how changes in these factors impact mortality rates.

Traditional statistical methods, which are primarily explanatory, face challenges when the number of variables increases, complicating the relationships between independent and dependent variables [16, 17]. These methods necessitate clear relationships and hypotheses to be established a priori. Therefore, employing traditional statistics for such multidimensional analyses can be quite challenging, necessitating the search for a more suitable methodological approach.

Machine learning (ML) presents itself as a more adept tool for addressing these complexities. ML is a computational approach that automates the process of learning solutions or parameters from data, aiming to achieve an optimal solution without the need for explicit programming instructions on problem-solving [18]. Unlike traditional statistics, ML emphasizes prediction and is capable of handling a multitude of variables, even in the absence of strict control over data collection or when dealing with non-linear interactions [16, 17, 19].

This study utilizes the Chinese Longitudinal Healthy Longevity Survey, a rich and representative dataset, to apply machine learning techniques. The aim is to explore the predictive power of various factors on older adults 4-year all-cause mortality in China and to develop a simplified ML model with robust predictive performance.

## Methods

### Data source and participants

Data for this study were sourced from the Chinese Longitudinal Healthy Longevity Survey (CLHLS) [20], a representative dataset of China’s older adults population conducted by the Research Center for Healthy Aging and Development of Peking University/National Development Institute. The survey employed a multi-stage, stratified, and targeted random sampling approach, selecting half of the counties and cities across 23 of China’s 31 provinces, excluding eight provinces in the western and northwestern regions due to less reliable age reporting among the older adults from ethnic minority groups [21, 22]. The CLHLS focuses on residents within the selected areas, conducting household interviews and recording current residences [22].

**Fig. 1:**
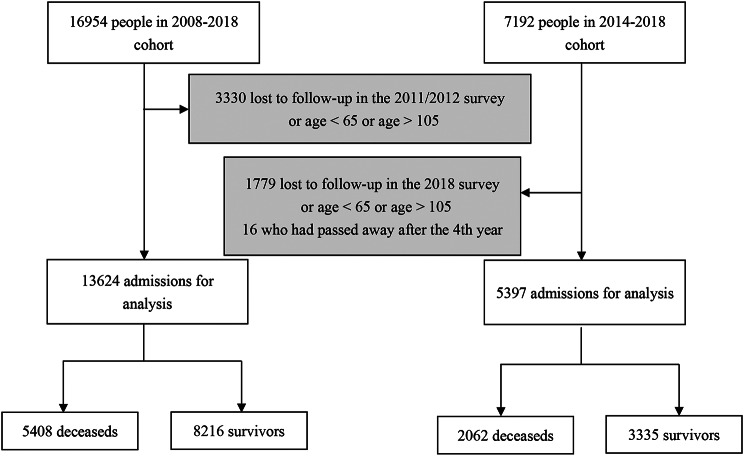
Flow chart

In our analysis, we used two cohorts from the CLHLS: one spanning from 2008 to 2018, initially comprising 16,954 participants, and another from 2014 to 2018, with 7,192 participants. The 2008–2018 cohort was employed to develop the models, whereas the 2014–2018 cohort was used for temporal validation. During the 2011/2012 survey, we observed a loss of 2,894 participants from the 2008–2018 cohort, and an additional 1,525 participants were lost in the 2014/2018 survey. After excluding participants who were lost to follow-up or did not meet the age criteria [23], the final sample sizes for analysis were 13,624 for the 2008–2018 cohort and 5,413 for the 2014–2018 cohort, as illustrated in Fig. 1.

Furthermore, responses marked as ‘not able to answer’ were treated as missing values [23] and were imputed using the mode. Categorical variables were encoded using one-hot encoding.

### Features and outcome

The 2008–2018 cohort of the CLHLS survey encompassed ten components: basic situation, life evaluation and personality, general ability, reaction ability, attention and calculation ability, recall, language, lifestyle, activities of daily living, personal background, and family structure.

Drawing from prior research on factors influencing mortality in older Chinese adults, we identified 16 key variables (features) for our analysis. These features encompassed a range of demographic and health-related variables, including sex, place of birth, education, marital status, economic status, smoking and drinking habits, exercise frequency, self-reported life satisfaction, self-reported health status, psychological resilience, cognitive function, the overall plant-diet index, leisure activities, activities of daily living, and instrumental activities of daily living. Each of these features is derived from the respective items within the CLHLS. A comprehensive list of these items, totaling 77 (Table S1) and consolidated them into the 16 key features (Table S2 and Table S3).

The primary outcome variable in our analysis was mortality, which was explicitly defined as a binary variable indicating the occurrence of 4-year all-cause mortality (yes/no) during the inter-survey periods. For the CLHLS 2008–2018 cohort, this period was between the 2008 survey and the subsequent 2011/2012 follow-up, whereas for the CLHLS 2014–2018 cohort, this period was between the 2014 survey and the subsequent 2018 survey follow-up.

#### Self-reported life satisfaction and self-reported health

In our study, self-reported life satisfaction and self-reported health were assessed using the questions, ‘How do you feel about your life right now?’ and ‘How do you feel about your own health status now?’ respectively. Participants provided their responses on a scale where ‘very good’ corresponded to 5 points, ‘good’ to 4 points, ‘so so’ to 3 points, ‘bad’ to 2 points, and ‘very bad’ to 1 point. A higher score indicates a higher level of life satisfaction or a perception of better health.

#### Psychological resilience

Psychological resilience was evaluated through seven specific items: ‘Do you often feel fearful or anxious?‘, ‘Do you often feel lonely and isolated?‘, ‘Do you feel the older you get, the more useless you are?‘, ‘Can you make your own decisions concerning your personal affairs?‘, ‘Do you always look on the bright side of things?‘, ‘To whom do you usually talk most frequently in daily life?‘, and ‘Who do you ask first for help when you have problems or difficulties?’ [10]. The first five items were responded to using a scale of ‘always’, ‘often’, ‘sometimes’, ‘seldom’, and ‘never’. Negative statements were scored from 0 to 4 points, whereas positive statements were scored in reverse, from 4 to 0 points. The items ‘Who do you usually talk to the most?’ and ‘If you have a concern or an idea, who do you talk to first?’ were scored with 2 points for having someone to talk to, 1 point for selecting ‘no one’, and 0 points for no one. The total score ranged from 0 to 22 points, with a higher score indicating greater psychological resilience.

#### Cognitive function

Cognitive function was assessed using the Chinese version of the Mini-Mental State Examination (MMSE) [24], a standardized tool that consists of five parts: orientation, registration, attention, calculation, and recall and language. Each question on the MMSE is scored as either ‘correct’ (1 point) or ‘wrong’ (0 points). The total score ranges from 0 to 30 points, with a higher score indicating stronger cognitive ability.

#### Plant-diet index (PDI)

The PDI was calculated with positive weightings for plant foods and negative weightings for animal foods [25]. Our assessment included 15 types of foods, categorized into plant foods—such as grains, fresh fruits, fresh vegetables, vegetable oils, legumes, garlic, nuts, tea, preserved vegetables, and sugar—and animal foods, which included animal fats, milk and dairy products, eggs, fish, and meat. The frequency of consumption for beans, garlic, nuts, tea, preserved vegetables, sugar, eggs, fish, meat, and milk and dairy products was rated on a scale from ‘almost everyday’ to ‘rarely or never,’ corresponding to 5 to 1 points, respectively. For fresh fruits and fresh vegetables, the scale was ‘every day/almost every day’ to ‘rarely or never,’ with scores ranging from 5 to 1 points. The intake of grains and vegetable oils was classified into a binary scoring system of 5 points for consumption and 1 point for non-consumption. The total PDI score ranges from 15 to 75, with a higher score indicating a more frequent intake of plant-based foods.

#### Leisure activities

Leisure activities were assessed through eight distinct items: housework, outdoor activities, planting flowers and raising birds, reading newspapers/surfing the Internet, raising poultry and livestock, playing cards or mahjong, watching TV and listening to the radio, and participating in social activities. Participants indicated the frequency of their engagement with each activity using the responses ‘almost every day’, ‘at least once a week’, ‘at least once a month’, ‘sometimes’, and ‘never’, which were assigned points on a scale from 1 to 5, respectively. The total leisure activity score ranges from 8 to 40, with higher scores indicating a higher frequency of engagement in leisure activities.

#### Activities of daily living (ADLs) and instrumental activities of daily living (IADLs)

ADLs encompass six essential daily activities: bathing, dressing, using the toilet, indoor transferring, maintaining continence, and eating. To further assess everyday functional competence, the concept of IADLs was introduced [26]. IADLs includes tasks such as visiting neighbors, shopping, cooking, washing clothes, walking two miles consecutively, lifting heavy objects weighing about 10 kg, performing squats and standing up three times consecutively, and using public transportation.

For both ADLs and IADLs assessments, participants were asked whether they could perform each task with responses categorized as ‘can’, ‘have some difficulty’, or ‘cannot’. These responses were then scored as 1, 2, and 3 points, respectively. The total score for ADL and IADL ranges from 14 to 42 points, with higher scores indicating a lower level of physical function.

#### Mortality

In the CLHLS, 4-year all-cause mortality were officially recorded upon confirmation through investigation. The date of death was verified using either a death certificate or confirmation from a local neighborhood committee [22]. For the CLHLS 2008–2018 cohort, the confirmation of older adults deaths was conducted during the 2011/2012 survey, while for the CLHLS 2014–2018 cohort, it was confirmed in the 2018 survey.

### Model development and performance

Initially, we developed models using 5-fold cross-validation. Our analysis incorporated five machine learning algorithms: Elastic-Net Regression (ENR), Decision Tree (DT), Random Forest (RF), K-Nearest Neighbor (KNN), and eXtreme Gradient Boosting (XGBoost). XGBoost, a distributed gradient boosting method, is favored by data scientists for its optimization capabilities and is widely used to achieve superior predictive performance [27]. We determined the optimal hyperparameters for each algorithm using a random grid search, with the hyperparameter range set to the default search space as provided by the *mlr3tuningspace* package (refer to Tables S4 and S5 for details).

Subsequently, we employed 5-fold cross-validation to assess the internal performance of the fitted models. Additionally, temporal validation was conducted using the CLHLS 2014–2018 cohort. More importantly, we have also employed the more straightforward, and simpler logistic regression (LR) algorithm as a baseline to compare the performance of other algorithms. The model exhibiting the best performance was analyzed using the *DALEX* package [28] to interpret variable importance and to generate local dependency plots. These plots illustrate the marginal effect of each feature on the machine learning model’s predictive outcomes. Based on the interpretation results, a subset of features with the greatest importance was identified for refitting the model and constructing a more streamlined version.

**Fig. 2:**
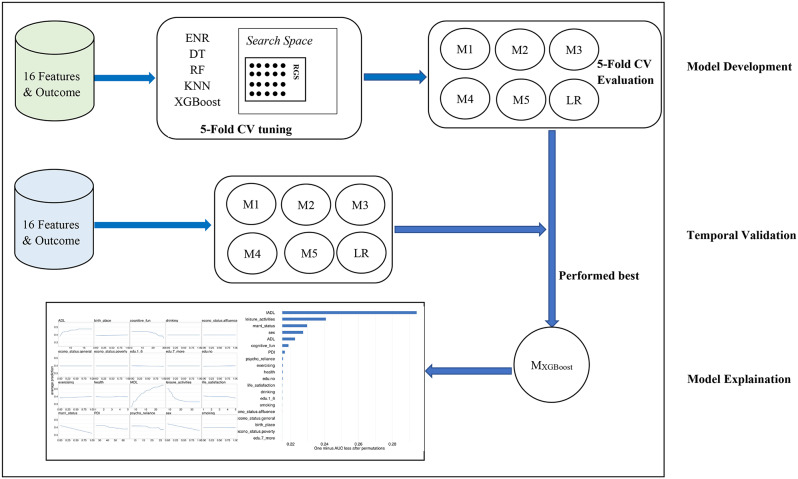
Models development, validation and explaination processes. We developed our predictive model using data from the 2008–2018 cohort (green in the Figure), with the 16 Features sourced from the 2008 survey and the Outcome ascertained from the 2011/2012 survey. Our analysis was conducted using five sophisticated machine learning algorithms: ENR, DT, RF, KNN, and XGBoost. Hyperparameter tuning for these algorithms was performed using the Random Grid Search (RGS) method and search space from the *mlr3tuningspace* package, which was evaluted 20 times with a 5-fold cross-validation (CV) process for each algorithm.The five models (M1-M5) were subsequently validated internally through another round of 5-fold CV to assess their performance. For temporal validation, we utilized data from the 2014–2018 cohort (blue in the Figure), with 16 features extracted from the 2014 survey and the outcome determined from the 2018 survey. Additionally, we used a no tuning LR as a baseline to compare with the aforementioned five algorithms. After a comprehensive evaluation that combined the results of internal and external validations, we identified the XGBoost-built model as the top performer. We then proceeded to explain the model in detail. Finally, based on the model explanation results, we extracted the eight most influential features and redeveloped the model for further validation. Ultimately, we chose the model developed using XGBoost for in-depth model explanation and analysis (The process is the same as the one depicted in the diagram, except that the XGBoost model is directly chosen for interpretation)

The area under the receiver operating characteristic (AUC) curve was utilized as a metric for discrimination [29]. We also utilized six additional evaluation metrics to compare the performance of the machine learning models: accuracy, sensitivity, specificity, positive predictive value (PPV), negative predictive value (NPV), and the F1 score. All analyses were conducted using R version 4.2.3, with machine learning analyses performed using the *mlr3* package [30]. The model development, validation and explanation processes can be seen in Fig. 2.

## Results

We developed our predictive model using the 2008–2018 cohort, wherein 16 features extracted from the 2008 survey data were utilized to predict mortality outcomes observed in the 2011/2012 survey. Concurrently, the 2014–2018 cohort served as the basis for temporal validation. Here, 16 features derived from the 2014 survey were employed to forecast whether the elderly participants would pass away, as indicated in the 2018 survey.The performance of the fitted models for internal validation and temporal validation is presented in Table 1.

**Table 1:** Performance comparison: internal and temporal validation of five models and logistic regression as a baseline with 16 and 8 features

Learner	AUC		Accuracy		Sensitivity		Specificity		PPV		NPV		F1	
	**16F**	**8F**	**16F**	**8F**	**16F**	**8F**	**16F**	**8F**	**16F**	**8F**	**16F**	**8F**	**16F**	**8F**
**Internal validation**														
LR	0.786	0.785	0.725	0.725	0.574	0.572	0.824	0.826	0.683	0.684	0.746	0.746	0.623	0.623
ENR	0.785	0.785	0.725	0.725	0.567	0.570	0.828	0.828	0.685	0.686	0.744	0.745	0.621	0.622
DT	0.763	0.758	0.711	0.715	0.594	0.602	0.788	0.789	0.649	0.653	0.747	0.751	0.620	0.626
RF	0.786	0.786	0.726	0.724	0.572	0.546	0.827	0.841	0.686	0.693	0.746	0.738	0.623	0.611
KNN	0.764	0.773	0.712	0.721	0.541	0.584	0.824	0.811	0.670	0.671	0.732	0.748	0.598	0.624
XGBoost	0.788	0.789	0.729	0.727	0.597	0.596	0.816	0.813	0.682	0.677	0.755	0.753	0.637	0.634
**Temporal validation**														
LR	0.807	0.807	0.755	0.755	0.516	0.519	0.903	0.901	0.767	0.764	0.751	0.752	0.617	0.618
ENR	0.807	0.807	0.754	0.754	0.508	0.516	0.906	0.902	0.769	0.764	0.749	0.751	0.612	0.616
DT	0.778	0.786	0.742	0.738	0.533	0.532	0.871	0.865	0.719	0.710	0.751	0.750	0.612	0.608
RF	0.802	0.802	0.748	0.744	0.489	0.459	0.908	0.921	0.767	0.782	0.742	0.734	0.598	0.579
KNN	0.784	0.785	0.746	0.746	0.481	0.515	0.910	0.889	0.768	0.741	0.739	0.748	0.591	0.608
XGBoost	0.806	0.806	0.751	0.752	0.527	0.510	0.890	0.902	0.748	0.763	0.753	0.749	0.618	0.611

*Note*. 16 F = 16 Features, 8 F = 8 Features. In addition, AUC = Area Under the Curve; PPV = Positive Predictive Value; NPV = Negative Predictive Value; F1 = F1 Score; LR = Logistic Regression; ENR = Elastic Net Regression; DT = Decision Tree; RF = Random Forest; KNN = K-Nearest Neighbor; XGBoost = eXtreme Gradient Boosting

In the internal validation phase, the XGBoost algorithm demonstrated the highest performance with an AUC of 0.788. The AUC values for LR, ENR, DT, RF, and KNN were 0.786, 0.785, 0.763, 0.786, and 0.764, respectively. For temporal validation, LR and ENR showed the best performance with an AUC of 0.807, closely followed by XGBoost with an AUC of 0.806. The AUC values for DT, RF, and KNN were 0.778, 0.802, and 0.784, respectively.

**Fig. 3:**
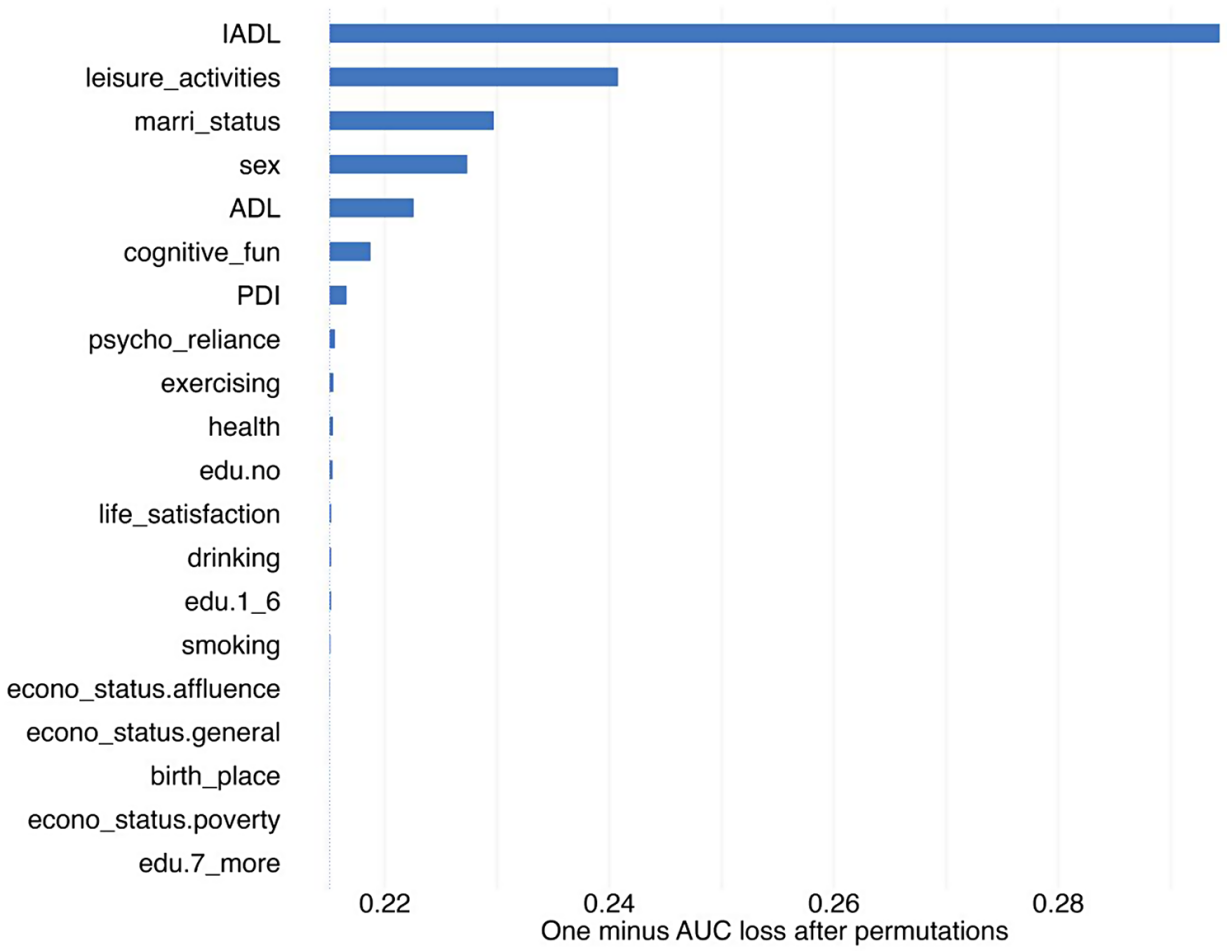
Variable Importance of XGBoost-Built Model Predicting 4-Year All-Cause Mortality in Older Adults with 16 Features. Note. IADL = Instrumental Activity of Daily Living; leisure_activities = leisure activities; marri_status = marital status; ADL = Activity of Daily Living; cognitive_fun = cognitive function; PDI = Plant-Diet Index; psycho_reliance = psychological resilience. For additional explanations regarding the titles, please see Supplementary Material, Table S3. The same below

**Fig. 4:**
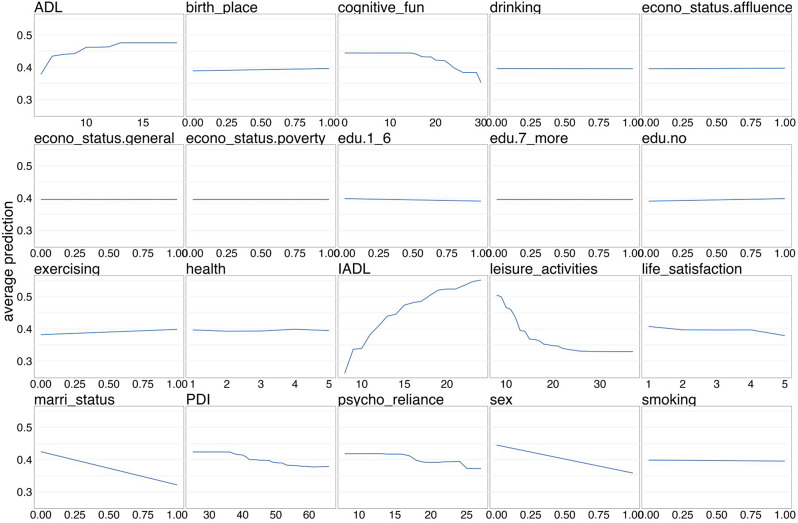
Partial dependence plots for the XGBoost-built model predicting 4-year all-cause mortality in older adults with 16 features. Note. During the data preprocessing phase, we implemented a hot-encoding procedure. Concurrently, for the variables of ADL and IADL, higher scores denote a lower level of physical functioning

After integrating the performance results from both internal validation and temporal validation, the XGBoost model was consequently selected for further interpretation. In the XGBoost model, the most influential variables identified were IADL, leisure activities, marital status, sex, ADL, cognitive function, PDI, and psychological resilience, as depicted in Fig. 3. The partial dependence plots indicated that higher scores in ADL and IADL, less frequent engagement in leisure activities, being unmarried, male gender, lower cognitive function, lower PDI, and weaker psychological resilience were associated with increased average predictions, as illustrated in Fig. 4.

Utilizing these significant 8 features, simpler models were built. At the same time, our concurrent internal and temporal validation results indicate that the performance of the simplified model is almost equivalent to that of the model prior to simplification.

## Discussion

To the best of our knowledge, our study represents the inaugural application of ML techniques to conduct a comprehensive analysis of the myriad factors influencing longevity among the older adults. This approach is akin to a ML-assisted meta-analysis, offering a sophisticated and data-driven synthesis of the existing literature on longevity in older individuals. We initially considered 16 distinct factors that could impact the mortality of older adults. Through this analysis, we identified the eight most influential features: IADLs, leisure activity, marital status, sex, ADLs, cognitive function, PDI, and psychological resilience. Based on these features, we developed a simplified machine learning model designed to predict longevity.

Besides, the model demonstrated strong predictive and generalization capabilities, particularly effective at forecasting the risk of death within a 4-year period for China’s older adults population. This more recent time of death prediction is considered significant for several reasons [31, 32]. Firstly, given the multitude of competing causes of death in the older adults, including the high incidence of cancer, a 4-year period is deemed more reflective of the likely changes in an older person’s health status [33]. Secondly, a shorter time frame enhances the accuracy of the model and facilitates the assessment of potential evidence-based interventions [32]. Additionally, it aligns with the preferences of older adults who tend to prioritize immediate quality of life over distant future events [34].

At the same time, partial dependence plots revealed that higher ADL and IADL scores, less frequent leisure activities, lower cognitive function, lower PDI, and lower psychological resilience were associated with increased average predictions, corroborating previous research [10–15]. ADLs refer to the ability to perform basic self-care tasks essential for survival, such as eating and dressing [35]. These skills are fundamental for an individual’s independence. IADLs, a related but distinct concept, pertain to the ability to carry out more complex daily activities necessary for living independently within a home and community setting. Examples of IADL include shopping and housework, which necessitate more sophisticated interactions with the environment [35]. Furthermore, IADL is linked to more intricate bodily functions and cognitive abilities [36].

ADLs and IADLs are crucial risk factors in predicting mortality among the older adults. Even after adjusting for other risk factors like age and the presence of cancer, the impairment of ADLs and IADLs remains a strong predictor of death, with more severe impairments correlating to a higher short-term risk of mortality [37]. Notably, the decline in ADLs becomes particularly pronounced in the four years preceding death [38]. While both ADLs and IADLs are significant, impairment in IADLs often occurs before that in ADLs [39]. Impairment in IADLs signifies more than just physical and cognitive limitations; it also implies a curtailment of social participation. Consequently, IADLs have a profound and wide-ranging impact on an individual’s quality of life and longevity.

Leisure activities have been shown to have a profound impact on the physiological mechanisms of the older adults, influencing the immune system, endocrine balance, and the central nervous system [40]. They can also affect the multi-system biological responses, thereby contributing to the overall health of older adults. Engagement in leisure activities has been proven to effectively reduce the prevalence of certain physical diseases, including cardiovascular disease [41] and chronic pain [42]. Moreover, leisure activities play a significant role in mental health, helping to alleviate loneliness [43], improve self-esteem, and decrease the incidence of depression [44]. A meta-analysis has even indicated that older adults who regularly engage in leisure activities have a 19% lower risk of death compared to those with little or no participation [45]. Given these benefits, it is crucial for communities to take proactive steps to promote leisure activities among the older adults. This could include improving sports facilities, organizing recreational events, and providing a variety of activities to cater to different interests and abilities.

Globally, women tend to outlive men, a phenomenon observed across nearly every nation and even in some other species [46]. This disparity can be attributed to a variety of biological and behavioral factors [47]. Biologically, women’s possession of two X chromosomes allows for a natural redundancy that can counteract the effects of harmful mutations, a biological advantage men do not share. Additionally, women may have a more robust immune response compared to men, and men might be more susceptible to adverse genetic mutations inherited from the mitochondrial DNA passed down from mothers. Behaviorally, men are more inclined to engage in risky behaviors, which can increase their likelihood of injury or death. Moreover, gender differences in health can also stem from variations in occupational hazards, family roles, and access to social welfare. Given these factors, it is essential for men to be more proactive about their personal health.

Cognitive functions, including attention, perception, speech, and language, can be impaired by factors such as aging and environmental influences [48]. Cognitive decline is not only a significant factor in the mortality of older individuals but may also serve as an indicator of the aging process [49]. Conditions such as high blood pressure, diabetes, and smoking are known to contribute to cognitive decline [50], which in turn can indirectly diminish the life expectancy of older adults. For instance, research has shown that high blood pressure, a condition affecting approximately two-thirds of older adults, significantly raises the risk of cognitive impairment and Alzheimer’s disease [51]. Consequently, the deterioration of cognitive abilities may signal the onset of certain diseases and could be an ominous harbinger of the end of life [49].

The PDI is a metric that favors diets rich in plant foods and low in animal foods [52]. A healthy plant-based diet is beneficial not only because it is typically high in dietary fiber and unsaturated fatty acids, which can help regulate metabolism and reduce the incidence of chronic diseases, but also because certain plant-based components promote intestinal health. Conversely, an unhealthy plant-based diet that is high in sodium or sugar can increase the risk of chronic diseases [14]. Making a transition from an animal-based to a plant-based diet has been shown to be cardiometabolically beneficial and is associated with a reduced likelihood of death [53]. For instance, a 10-point increase in PDI scores has been correlated with a 7% decrease in the risk of cardiovascular disease mortality [52]. Thus, in today’s era of increasing health consciousness, the adoption of a plant-based diet is recommended as a positive step towards better health.

Finally, marital status and psychological resilience are significant factors that influence mortality among older adults. Those who are married tend to enjoy better health and have a longer life expectancy than those without a spouse [54]. Additionally, individuals with high psychological resilience—a measure of positive adaptation to adversity—experience a 20% lower risk of all-cause mortality compared to those with lower resilience [55]. Beyond these factors, other aspects such as smoking, alcohol consumption, self-reported life satisfaction, physical activity levels, education, economic status, and self-reported health are also known to impact mortality in older adults. However, in this study, their effects were found to be relatively minor. It is possible that these factors may exert their influence on mortality in older adults through their interaction with more significant factors, such as ADLs.

Furthermore, the model demonstrated robust performance in temporal validation, indicating strong generalization capabilities and a lower risk of overfitting. Additionally, the model’s performance in temporal validation appears to be “better” compared to its performance in internal validation, and there are many reasons for this. Firstly, it may be because the data distribution used in internal validation differs from that used in temporal validation, and this discrepancy makes the model’s performance " better” in temporal validation. Secondly, it could also be due to the influence of randomness. The training and validation process of the machine learning model is subject to random factors, such as random data partitioning or random initialization of parameters. This randomness can make the performance of the machine learning model on the temporal validation set appear “better”. Lastly, during the training process, the machine learning model may have learned additional “knowledge” that is more relevant to the temporal validation dataset, thereby leading to “better” performance in temporal validation. However, this apparent better performance in temporal validation compared to internal validation does not completely rule out the influence of randomness. Nevertheless, it is most importantly indicative of our model’s strong generalization ability, lending strong credibility to the results.

Notably, The simplified models developed using ENR, DT, RF, KNN, and XGBoost exhibit performance that is largely comparable to the models prior to simplification, which suggests that the model simplification was successful. In aggregate, the mean AUC for models built using six different algorithms—LR (as a baseline), ENR, DT, RF, KNN, and XGBoost—were as follows: 0.796, 0.796 for ENR, 0.771 for DT, 0.794 for RF, 0.777 for KNN, and 0.797 for XGBoost. The corresponding standard deviations were 0.012, 0.013, 0.013, 0.009, 0.010, and 0.010, respectively. Given the performance and stability of the models, we recommend the XGBoost model for future applications due to its consistent and reliable predictive accuracy.

Additionally, although LR is considered a simpler algorithm within the realm of ML, its performance in this study is by no means second-rate. In our research, the performance of LR was on par with ENR and even competes with the more complex ensemble algorithm, XGBoost. This could be due to the limited number of features used, which have relatively uncomplicated relationships. It illustrates the advantage of applying LR in scenarios with a smaller set of features. After all, LR operates with fewer resource demands and at a faster pace compared to XGBoost. This provides some enlightenment for ML research: it is appropriate to consider simpler algorithms rather than relentlessly pursuing more complex ones. The relative strengths and weaknesses of different algorithms can vary under different circumstances, as encapsulated by the saying “there is no free lunch,” which necessitates careful deliberation in specific contexts [56].

There are several limitations to this study. Firstly, the feature set used in our models did not include some key variables known to affect the longevity of the older adults, such as various diseases, which did not contain in the survey. Incorporating these factors could potentially enhance the performance of our models. Secondly, our data was exclusively derived from a Chinese population, which may limit the generalizability of our findings. Future research should aim to include more diverse, cross-cultural groups to enhance the external validity of the models. Thirdly, the reliance on self-reported data introduces potential biases, such as respondents providing random answers without fully understanding the questionnaire or the influence of social desirability bias. Lastly, while women and men have different biological characteristics, they were analyzed together in this study. Our primary goal was to construct and explain a mortality prediction model for the overall older adults population, which aligns with the current social context where gender differences in longevity are less commonly distinguished. However, we recognize that gender-specific analyses may reveal new insights, and this is an aspect we intend to explore in future research, pending the scope and length of the study.

## Conclusions

Our study represents a novel application of machine learning to comprehensively analyze factors influencing longevity among older adults. By focusing on a simplified model that incorporates key features such as IADL, leisure activity, marital status, sex, ADL, cognitive function, PDI, and psychological resilience, we have demonstrated the potential to predict 4-year all-cause mortality risk with high accuracy. The XGBoost model, in particular, emerged as a reliable predictor of mortality, making it a suitable choice for future applications. Our study provides valuable insights into the complex interplay of factors that contribute to the longevity of older adults. The development of a predictive model that can accurately forecast mortality risk is a significant step towards enhancing healthcare interventions for the older adults. By identifying the most influential factors and understanding their impact, we can better tailor our approaches to promote healthy aging and improve the quality of life for older adults.

### Electronic supplementary material

Below is the link to the electronic supplementary material.

Supplementary Material 1

## Data Availability

The datasets used and/or analyzed during the current study are available anytime from the corresponding author on reasonable request.

## Abbreviations

CLHLS: Chinese Longitudinal Healthy Longevity Survey
LR: Logistic Regression
ENR: Elastic Net Regression
DT: Decision Tree
RF: Random Forest
KNN: K-Nearest Neighbor
XGBoost: eXtreme Gradient Boosting
AUC: Area under the receiver operating characteristic
PPV: Positive predictive value
NPV: Negative predictive value
MMSE: Mini-Mental State Examination
IADL: Instrumental activity of daily living
ADL: Activity of daily living
PDI: Overall plant-based diet index
