# Supplementary material for: What factors preventing the older adults in China from living longer: a machine learning study
Source: BMC Geriatr. 2024 Jul 22;24:625. doi: 10.1186/s12877-024-05214-8 (PMC11265125; doi:10.1186/s12877-024-05214-8)
Supplement: Supplementary file 1 — Supplementary Material 1 [file 12877_2024_5214_MOESM1_ESM.docx]

**Supplementary Materials**

**Content**

[**Table S1.** Patterns of ‘Not able to answer’, ‘Missing’, and ‘NA’ Data Across Two Cohorts: 2008- 2018 and 2014-2018…...………………………………………………………………1](#T1)

[**Table S2.** 77 CLHLS Questionnaire Items and the Corresponding 16 Features……………………..…………………………………………………………..…….6](#T2)

[**Table S3.** Descriptive Analysis of Cohorts: 2008-2018 and 2014-2018………………………………..…….……………………………………………………9](#T3)

[**Table S4.** Hyperparameter Tuning Results for Elastic Net Regression (ENR), Decision Tree (DT), Random Forest (RF), K-Nearest Neighbor (KNN) and eXtreme Gradient Boosting (XGBoost) with 16 Features……………………………….………………………………...11](#T4)

[**Table S5.** Hyperparameter Tuning Results for ENR, DT, RF, KNN and XGBoost with 8 Features………………………………………………………………………………………12](#T5)

[**Figure S1.** Variable Importance of XGBoost-Built Model Predicting 4-Year All-Cause Mortality. in Older Adults with 8 Features………..………………………………………....13](#F1)

**[Figure S2.](#F2)** [Partial Dependence Plots for the XGBoost-Built Model Predicting 4-Year All-Cause Mortality in Older Adults with 8 Features……………………………………………14](#F2)

**Table S1.** Patterns of ‘Not able to answer’, ‘Missing’, and ‘NA’ Data Across Two Cohorts: 2008- 2018 and 2014-2018

| **Number** | **Description** | **Not able to answer (%)** | |  | **Missing^b^**  **(%)** | |  | **NA^c^**  **(%)** | |
| --- | --- | --- | --- | --- | --- | --- | --- | --- | --- |
|  |  | **2008^a^** | **2014^a^** |  | **2008** | **2014** |  | **2008** | **2014** |
| **Basic information** | | | | | | | | | |
| 1 | Sex | 0 | 0 |  | 0 | 0 |  | 0 | 0 |
| 2 | Was the place of birth an urban area or a rural area at time of birth? | 5  (0.04%) | 2  (0.04%) |  | 7  (0.05%) | 1  (0.02%) |  | 4  (0.03%) | 441  (8.17%) |
| **Life evaluation and personality** | | | | | | | | | |
| 3 | How do you feel about your life right now? | 1626  (11.93%) | 336  (6.23%) |  | 0 | 1  (0.02%) |  | 0 | 60  (1.11%) |
| 4 | How do you feel about your own health status now? | 1624  (11.92%) | 336  (6.23%) |  | 0 | 1  (0.02%) |  | 0 | 62  (1.15%) |
| 5 | Do you always look on the bright side of things? | 1865  (13.69%) | 436  (8.08%) |  | 0 | 1  (0.02%) |  | 0 | 60  (1.11%) |
| 6 | Do you often feel fearful or anxious? | 1926  (14.14%) | 500  (9.26%) |  | 0 | 1  (0.02%) |  | 0 | 61  (1.13%) |
| 7 | Do you often feel lonely and isolated? | 1948  (14.30%) | 474  (8.78%) |  | 0 | 1  (0.02%) |  | 0 | 64  (1.19%) |
| 8 | Can you make your own decisions concerning your personal affairs? | 2009  (14.75%) | 549  (10.17%) |  | 0 | 1  (0.02%) |  | 0 | 66  (1.22%) |
| 9 | Do you feel the older you get, the more useless you are? | 2040  (14.97%) | 517  (9.58%) |  | 0 | 1  (0.02%) |  | 0 | 65  (1.20%) |
| **General ability** | | | | | | | | | |
| 10 | What time of day is it right now (morning, afternoon, evening)? | 1958  (14.37%) | 480  (8.89%) |  | 0 | 1  (0.02%) |  | 0 | 64  (1.19%) |
| 11 | What is the animal year of this year? | 2133  (15.66%) | 614  (11.38%) |  | 0 | 1  (0.02%) |  | 0 | 64  (1.19%) |
| 12 | What is the date (Chinese calendar day and month) of the mid-autumn festival? | 2196  (16.12%) | 646  (11.97%) |  | 0 | 1  (0.02%) |  | 0 | 65  (1.20%) |
| 13 | What is the season right now, spring, summer, fall, winter? | 2046  (15.02%) | 575  (10.65%) |  | 0 | 1  (0.02%) |  | 0 | 65  (1.20%) |
| 14 | What is the name of this district or town? | 2104  (15.44%) | 568  (10.52%) |  | 0 | 1  (0.02%) |  | 0 | 72  (1.33%) |
| 15 | Please nameas many kinds of food as possible in 1 minute. | 1443  (10.59%) | 124  (2.30%) |  | 70  (0.51%) | 14  (0.26%) |  | 0 | 158  (2.93%) |
| **Reaction capacity** | | | | | | | | | |
| 16 | Repeat the name of “table” at first attempt | 2405  (17.65%) | 655  (12.14%) |  | 0 | 1  (0.02%) |  | 0 | 67  (1.24%) |
| 17 | Repeat the name of “apple”at first attempt | 2412  (17.70%) | 656  (12.15%) |  | 0 | 1  (0.02%) |  | 0 | 98  (1.82%) |
| 18 | Repeat the name of “clothes”at first attempt | 2437  (17.89%) | 668  (12.38%) |  | 0 | 1  (0.02%) |  | 0 | 121  (2.24%) |
| **Attention and calculation ability** | | | | | | | | | |
| 19 | $20-$3=? | 2653  (19.47%) | 800  (14.82%) |  | 0 | 1  (0.02%) |  | 0 | 65  (1.20%) |
| 20 | $20-$3-$3=? | 2773  (20.35%) | 844  (15.64%) |  | 0 | 1  (0.02%) |  | 0 | 68  (1.26%) |
| 21 | $20-$3-$3-$3=? | 2856  (20.96%) | 888  (16.45%) |  | 0 | 1  (0.02%) |  | 0 | 68  (1.26%) |
| 22 | $20-$3-$3-$3-$3=? | 2915  (21.40%) | 921  (17.07%) |  | 0 | 1  (0.02%) |  | 0 | 72  (1.33%) |
| 23 | $20-$3-$3-$3-$3-$3=? | 2942  (21.59%) | 940  (17.42%) |  | 0 | 1  (0.02%) |  | 0 | 110  (2.04%) |
| 24 | Draw the figure | 5092  (37.38%) | 1675  (31.04%) |  | 2521  (18.50%) | 740  (13.71%) |  |  | 149  (2.76%) |
| **Recall** | | | | | | | | | |
| 25 | Repeat the name of “table” a while later | 2847  (20.90%) | 896  (16.60%) |  | 0 | 1  (0.02%) |  | 0 | 65  (1.20%) |
| 26 | Repeat the name of “apple” a while later | 2876  (21.11%) | 907  (16.81%) |  | 0 | 1  (0.02%) |  | 0 | 74  (1.37%) |
| 27 | Repeat the name of “clothes” while later | 2891  (21.22%) | 935  (17.32%) |  | 0 | 1  (0.02%) |  | 0 | 73  (1.35%) |
| **Language** | | | | | | | | | |
| 28 | Name “pen” | 2089  (15.33%) | 571  (10.58%) |  | 0 | 1  (0.02%) |  | 0 | 67  (1.24%) |
| 29 | Name “watch” | 2116  (15.53%) | 582  (10.78%) |  | 0 | 1  (0.02%) |  | 0 | 74  (1.37%) |
| 30 | Repeat a sentence | 2728  (20.02%) | 748  (13.86%) |  | 0 | 1  (0.02%) |  | 0 | 84  (1.56%) |
| 31 | Taking paper using right hand | 2580  (18.94%) | 764  (14.16%) |  | 0 | 2  (0.04%) |  | 0 | 70  (1.30%) |
| 32 | Fold paper | 2791  (20.49%) | 834  (15.45%) |  | 0 | 2  (0.04%) |  | 0 | 71  (1.32%) |
| 33 | Put paper on the floor | 2868  (21.05%) | 882  (16.34%) |  | 0 | 2  (0.04%) |  | 0 | 71  (1.32%) |
| **Lifestyle** | | | | | | | | | |
| 34 | Please tell us the staple food you eat | 0 | 0 |  | 0 | 0 |  | 0 | 56  (1.04%) |
| 35 | How often eat fresh fruit? | 0 | 1  (0.02%) |  | 1  (0.01%) | 0 |  | 0 | 60  (1.11%) |
| 36 | How often eat vegetables? | 1  (0.01%) | 1  (0.02%) |  | 1  (0.01%) | 0 |  | 0 | 60  (1.11%) |
| 37 | What kind of grease do you mainly use for cooking | 1  (0.01%) | 2  (0.04%) |  | 1  (0.01%) | 6  (0.11%) |  | 1  (0.01%) | 85  (1.57%) |
| 38 | How often eat meat at present? | 9  (0.07%) | 1  (0.02%) |  | 0 | 0 |  | 0 | 92  (1.70%) |
| 39 | How often eat fish at present? | 9  (0.07%) | 1  (0.02%) |  | 0 | 0 |  | 0 | 94  (1.74%) |
| 40 | How often eat egg at present? | 9  (0.07%) | 1  (0.02%) |  | 0 | 0 |  | 0 | 92  (1.70%) |
| 41 | How often eat bean at present? | 10  (0.07%) | 1  (0.02%) |  | 0 | 0 |  | 0 | 95  (1.76%) |
| 42 | How often eat salt-preserved vegetables at present? | 10  (0.07%) | 1  (0.02%) |  | 0 | 0 |  | 0 | 97  (1.80%) |
| 43 | How often eat sugar at present? | 9  (0.07%) | 1  (0.02%) |  | 0 | 0 |  | 0 | 97  (1.80%) |
| 44 | How often eat tea at present? | 9  (0.07%) | 1  (0.02%) |  | 0 | 8  (0.15%) |  | 0 | 99  (1.83%) |
| 45 | How often eat garlic at present? | 9  (0.07%) | 1  (0.02%) |  | 0 | 1  (0.02%) |  | 0 | 96  (1.78%) |
| 46 | How often eat milk products at present? | 9  (0.07%) | 1  (0.02%) |  | 0 | 0 |  | 0 | 97  (1.80%) |
| 47 | How often eat nut products at present? | 10  (0.07%) | 1  (0.02%) |  | 0 | 4  (0.07%) |  | 0 | 97  (1.80%) |
| 48 | Do you smoke at the present time? | 0 | 0 |  | 0 | 2  (0.04%) |  | 0 | 69  (1.28%) |
| 49 | Do you drink alcohol at the present time? | 0 | 0 |  | 0 | 0 |  | 0 | 93  (1.72%) |
| 50 | Do you do exercises regularly at present? | 0 | 2  (0.04%) |  | 0 | 3  (0.06%) |  | 0 | 166  (3.08%) |
| 51 | Do you do house work at present? | 0 | 0 |  | 0 | 0 |  | 0 | 67  (1.24%) |
| 52 | Do you grow vegetables & do other field work at present? | 0 | 0 |  | 0 | 0 |  | 0 | 65  (1.20%) |
| 53 | Do you do garden work? | 0 | 0 |  | 0 | 0 |  | 0 | 72  (1.33%) |
| 54 | Do you read newspapers/books at present? | 0 | 0 |  | 0 | 0 |  | 0 | 71  (1.32%) |
| 55 | Do you raise domestic animals/pets at present? | 0 | 0 |  | 0 | 0 |  | 0 | 73  (1.35%) |
| 56 | Do you play cards/mah-jongg at present? | 0 | 0 |  | 0 | 0 |  | 0 | 70  (1.30%) |
| 57 | Do you watch tv or listen to radio at present? | 0 | 0 |  | 0 | 0 |  | 0 | 67  (1.24%) |
| 58 | Do you take part. in some social activities at present? | 0 | 0 |  | 0 | 0 |  | 0 | 76  (1.41%) |
| **Activities of daily living** | | | | | | | | | |
| 59 | Feeding | 0 | 0 |  | 0 | 0 |  | 0 | 149  (2.76%) |
| 60 | Bathing | 0 | 0 |  | 1  (0.01%) | 7  (0.13%) |  | 0 | 80  (1.48%) |
| 61 | Dressing | 0 | 0 |  | 0 | 0 |  | 0 | 75  (1.39%) |
| 62 | Toileting | 0 | 0 |  | 0 | 0 |  | 0 | 74  (1.37%) |
| 63 | Indoor transferring | 0 | 0 |  | 0 | 1  (0.02%) |  | 0 | 85  (1.57%) |
| 64 | Continence | 0 | 0 |  | 0 | 0 |  | 0 | 75  (1.39%) |
| 65 | Able to go outside to visit neighbors? | 0 | 0 |  | 0 | 5  (0.09%) |  | 0 | 62  (1.15%) |
| 66 | Able to go shopping by self? | 0 | 0 |  | 0 | 4  (0.07%) |  | 0 | 61  (1.13%) |
| 67 | Able to make food by self? | 0 | 0 |  | 0 | 2  (0.04%) |  | 0 | 63  (1.16%) |
| 68 | Able to wash clothes by self? | 0 | 0 |  | 0 | 1  (0.02%) |  | 0 | 68  (1.26%) |
| 69 | Able to walk one kilometer? | 0 | 0 |  | 0 | 0 |  | 0 | 69  (1.28%) |
| 70 | Able to carry 5kg weight? | 0 | 0 |  | 0 | 0 |  | 0 | 64  (1.19%) |
| 71 | Able to crouch and stand three times? | 0 | 0 |  | 0 | 0 |  | 0 | 64  (1.19%) |
| 72 | Able to take public transportation? | 0 | 0 |  | 0 | 2  (0.04%) |  | 0 | 80  (1.48%) |
| **Personal background and family structure** | | | | | | | | | |
| 73 | How many years did you attend school? | 24  (0.18%) | 2  (0.04%) |  | 9  (0.07%) | 2  (0.04%) |  | 4  (0.03%) | 46  (0.85%) |
| 74 | How do you rate your economic status compared with others in your local area? | 32  (0.23%) | 42  (0.78%) |  | 0 | 0 |  | 0 | 73  (1.35%) |
| 75 | Current marital status | 0 | 0 |  | 0 | 1  (0.02%) |  | 0 | 110  (2.04%) |
| 76 | To whom do you usually talk most frequently in daily life? | 2  (0.02%) | 56  (1.04%) |  | 3  (0.02%) | 24  (0.44%) |  | 0 | 111  (2.06%) |
| 77 | Who do you first for help when you have problems/difficulties? | 4  (0.03%) | 42  (0.78%) |  | 3  (0.02%) | 15  (0.28%) |  | 0 | 2246  (41.62%) |

*^a^N_2008-2018 cohort_ = 13624; N_2014-2018 cohort_ = 5397; ^b^Missing refers to artificial missing values; NA refers to system missing value.*

We consider 'Not able to answer' as missing values. Moreover, for the imputation of missing values, we use the mode to fill the variables (or features) for both the 2008-2018 (for model development and internal validation) and 2014-2018 cohorts (for temporal validation) respectively.

**Table S2.** 77 CLHLS Questionnaire Items and the Corresponding 16 Features

| **Number of features** | **Variables/Features** | **Items** |
| --- | --- | --- |
| 1 | Sex | 1. Sex |
| 2 | Place of birth | 2. Was the place of birth an urban area or a rural area at time of birth? |
| 3 | Education | 3. How many years did you attend school? |
| 4 | Marital status | 4. Current marital status |
| 5 | Economic status | 5. How do you rate your economic status compared with other local people? |
| 6 | Smoking | 6. Do you smoke at the present time? |
| 7 | Drinking | 7. Do you drink alcohol at the present time? |
| 8 | Exercising | 8. Do you do exercises regularly at present? |
| 9 | Self-report life satisfaction | 9. How do you feel about your life right now? |
| 10 | Self-report health | 10. How do you feel about your own health status now? |
| 11 | Psychological resilience | 11. Do you often feel fearful or anxious? |
|  |  | 12. Do you often feel lonely and isolated? |
|  |  | 13. Do you feel the older you get, the more useless you are? |
|  |  | 14. Can you make your own decisions concerning your personal affairs? |
|  |  | 15. Do you always look on the bright side of things? |
|  |  | 16. To whom do you usually talk most frequently in daily life? |
|  |  | 17. Who do you ask first for help when you have problems or difficulties? |
| 12 | Cognitive function | 18. What time of day is it right now (morning, afternoon, evening)? |
|  |  | 19. What is the animal year of this year? |
|  |  | 20. What is the date (Chinese calendar day and month) of the mid-autumn festival? |
|  |  | 21. What is the season right now, spring, summer, fall, winter? |
|  |  | 22. What is the name of this district or town? |
|  |  | 23. Please nameas many kinds of food as possible in 1 minute. |
|  |  | 24. Repeat the name of "table" at first attempt |
|  |  | 25. Repeat the name of "apple" at first attempt |
|  |  | 26. Repeat the name of "clothes" at first attempt |
|  |  | 27. $20-$3=? |
|  |  | 28. $20-$3-$3=? |
|  |  | 29. $20-$3-$3-$3=? |
|  |  | 30. $20-$3-$3-$3-$3=? |
|  |  | 31. $20-$3-$3-$3-$3-$3=? |
|  |  | 32. Draw the figure |
|  |  | 33. Repeat the name of "table" a while later |
|  |  | 34. Repeat the name of "apple" a while later |
|  |  | 35. Repeat the name of "clothes" a while later |
|  |  | 36. Name "pen" |
|  |  | 37. Name "watch" |
|  |  | 38. Repeat a sentence |
|  |  | 39. Taking paper using right hand |
|  |  | 40. Fold paper |
|  |  | 41. Put paper on the floor |
| 13 | PDI | 42. Please tell us the staple food you eat |
|  |  | 43. How often eat fresh fruit? |
|  |  | 44. How often eat vegetables? |
|  |  | 45. What kind of grease do you mainly use for cooking? |
|  |  | 46. How often eat meat at present? |
|  |  | 47. How often eat fish at present? |
|  |  | 48. How often eat egg at present? |
|  |  | 49. How often eat bean at present? |
|  |  | 50. How often eat salt-preserved vegetables at present? |
|  |  | 51. How often eat sugar at present? |
|  |  | 52. How often eat tea at present? |
|  |  | 53. How often eat garlic at present? |
|  |  | 54. How often eat milk products at present? |
|  |  | 55. How often eat nut products at present? |
| 14 | Leisure acitivities | 56. Do you do house work at present? |
|  |  | 57. Do you grow vegetables & do other field work at present? |
|  |  | 58. Do you do garden work? |
|  |  | 59. Do you read newspapers/books at present? |
|  |  | 60. Do you raise domestic animals/pets at present? |
|  |  | 61. Do you play cards/mah-jongg at present? |
|  |  | 62. Do you watch tv or listen to radio at present? |
|  |  | 63. Do you take part in some social activities at present? |
| 15 | ADL | 64. Feeding |
|  |  | 65. Bathing |
|  |  | 66. Dressing |
|  |  | 67. Toileting |
|  |  | 68. Indoor transferring |
|  |  | 69. Continence |
| 16 | IADL | 70. Able to go outside to visit neighbors? |
|  |  | 71. Able to go shopping by self? |
|  |  | 72. Able to make food by self? |
|  |  | 73. Able to wash clothes by self? |
|  |  | 74. Able to walk one kilometer? |
|  |  | 75. Able to carry 5kg weight? |
|  |  | 76. Able to crouch and stand three times? |
|  |  | 77. Able to take public transportation? |

**Table S3.** Descriptive Analysis of Cohorts: 2008-2018 and 2014-2018

| **Number** | **Feature** | **Code** | **Type** | **2008** | |  | **2014** | |  | ***p*-value^c^** | |
| --- | --- | --- | --- | --- | --- | --- | --- | --- | --- | --- | --- |
|  |  |  |  | survivor | deceased |  | survivor | deceased |  | 2008 | 2014 |
| 1 | Sex | sex | Factor |  |  |  |  |  |  | <0.001 | 0.206 |
|  | Male | 0 |  | 3723**^b^**  (27.33%) | 2156  (15.83%) |  | 1602  (29.60%) | 954  (17.68%) |  |  |  |
|  | Female | 1 |  | 4493  (32.98%) | 3252  (23.87%) |  | 1733  (32.02%) | 1108 (20.53%) |  |  |  |
| 2 | Birth place | birth_place | Factor |  |  |  |  |  |  | 0.002 | 0.713 |
|  | Urban | 0 |  | 957  (7.02%) | 538  (3.95%) |  | 264  (4.88%) | 169 (3.13%) |  |  |  |
|  | Rural | 1 |  | 7259  (53.28%) | 4870  (35.75%) |  | 3071  (56.73%) | 1893 (35.08%) |  |  |  |
| 3 | Education | edu | Factor |  |  |  |  |  |  | <0.001 | <0.001 |
|  | No formal education | no |  | 5062  (37.16%) | 4049  (29.72%) |  | 1909  (35.27%) | 1418 (26.27%) |  |  |  |
|  | 1 to 6 | 1_6 |  | 2589  (19.00%) | 1143  (8.39%) |  | 1187  (21.93%) | 567 (10.51%) |  |  |  |
|  | 7 to more | 7_more |  | 565  (4.15%) | 216  (1.59%) |  | 239  (4.42%) | 77 (1.43%) |  |  |  |
| 4 | Marital status | marri_status | Factor |  |  |  |  |  |  | <0.001 | <0.001 |
|  | Without spouse | 0 |  | 4675  (34.31%) | 4457  (32.71%) |  | 1687  (31.17%) | 1528 (28.31%) |  |  |  |
|  | Have spouse | 1 |  | 3541  (25.99%) | 951  (6.98%) |  | 1648  (30.45%) | 534  (9.89%) |  |  |  |
| 5 | Economic status | econo_status | Factor |  |  |  |  |  |  | <0.001 | 0.245 |
|  | Affluence | affluence |  | 261  (1.92%) | 113  (0.83%) |  | 109  (2.01%) | 54  (1.00%) |  |  |  |
|  | General | general |  | 5548  (40.72%) | 3595  (26.38%) |  | 2422  (44.74%) | 1483  (27.48%) |  |  |  |
|  | Poverty | poverty |  | 2407  (17.67%) | 1700  (12.48%) |  | 804  (14.85%) | 525 (9.73%) |  |  |  |
| 6 | Smoking | smoking | Factor |  |  |  |  |  |  | <0.001 | <0.001 |
|  | Yes | 0 |  | 1654  (12.14%) | 789  (5.79%) |  | 588  (10.86%) | 274  (5.08%) |  |  |  |
|  | No | 1 |  | 6562  (48.17%) | 4619  (33.90%) |  | 2747  (50.75%) | 1788 (33.13%) |  |  |  |
| 7 | Drinking | drinking | Factor |  |  |  |  |  |  | <0.001 | <0.001 |
|  | Yes | 0 |  | 1615  (11.85%) | 812  (5.96%) |  | 567  (10.47%) | 250  (4.63%) |  |  |  |
|  | No | 1 |  | 6601  (48.45%) | 4596  (33.73%) |  | 2768  (51.14%) | 1812  (33.57%) |  |  |  |
| 8 | Exercising | exercising | Factor |  |  |  |  |  |  | <0.001 | <0.001 |
|  | Yes | 0 |  | 2732  (20.05%) | 998  (7.33%) |  | 1025  (18.94%) | 342  (6.34%) |  |  |  |
|  | No | 1 |  | 5484  (40.25%) | 4410  (32.37%) |  | 2310  (42.68%) | 1720  (31.87%) |  |  |  |
| 9 | Self-report life satisfaction | life_satisfaction | Numeric | 3.69  (0.80) | 3.68  (0.75) |  | 3.82  (0.76) | 3.78  (0.75) |  | 0.464 | 0.027 |
| 10 | Self-report health | health | Numeric | 3.53  (0.90) | 3.46  (0.88) |  | 3.43  (0.86) | 3.19  (0.82) |  | <0.001 | <0.001 |
| 11 | Psychological resilience | psycho_reliance | Numeric | 21.22  (3.06) | 20.70  (3.29) |  | 21.12  (2.84) | 20.32  (2.87) |  | <0.001 | <0.001 |
| 12 | Cognitive function | cognitive_fun | Numeric | 26.40  (4.20) | 24.40  (5.47) |  | 27.76  (2.99) | 25.93  (4.40) |  | <0.001 | <0.001 |
| 13 | PDI**^a^** | PDI | Numeric | 48.42  (6.08) | 46.78  (6.10) |  | 47.16  (5.61) | 45.67  (5.46) |  | <0.001 | <0.001 |
| 14 | Leisure acitivities | leisure_activities | Numeric | 18.60  (6.06) | 13.50  (5.48) |  | 20.18  (6.18) | 14.39  (6.06) |  | <0.001 | <0.001 |
| 15 | ADL**^a^** | ADL | Numeric | 6.34  (1.31) | 7.77  (3.07) |  | 6.24  (1.04) | 8.10  (3.33) |  | <0.001 | <0.001 |
| 16 | IADL**^a^** | IADL | Numeric | 11.75  (5.05) | 17.30  (5.99) |  | 10.65  (4.21) | 16.76  (6.26) |  | <0.001 | <0.001 |

***^a^****PDI = overall plant-based diet index, ADL = activity of daily living, IADL = instrumental activity of daily living;* ***^b^****Frequency (proportion)/ mean (standard deviation);* ***^c^****Chi-square test for factor type and t-test for numeric type.*

**Table S4.** Hyperparameter Tuning Results for Elastic Net Regression (ENR), Decision Tree (DT), Random Forest (RF), K-Nearest Neighbor (KNN) and eXtreme Gradient Boosting (XGBoost) with 16 Features

| **Learner** | **Parameter set** | **Search space** | **Parameter result** | | | | | **AUC** | | | | |
| --- | --- | --- | --- | --- | --- | --- | --- | --- | --- | --- | --- | --- |
| **CV** | | | **1** | **2** | **3** | **4** | **5** | **1** | **2** | **3** | **4** | **5** |
| ENR | s | 1e-04 to 10000 | 0.004072723 | 0.004360547 | 0.0004624161 | 0.007223278 | 0.02193468 | 0.782 | 0.785 | 0.786 | 0.784 | 0.787 |
|  | alpha | 0e+00 to 1 | 0.6471561 | 0.4780939 | 0.9199594 | 0.5645738 | 0.1785097 |  |  |  |  |  |
| DT | minsplit | 2e+00 to 128.0 | 81 | 4 | 2 | 21 | 4 | 0.766 | 0.763 | 0.761 | 0.763 | 0.763 |
|  | minbucket | 1e+00 to 64.0 | 44 | 34 | 62 | 57 | 55 |  |  |  |  |  |
|  | cp | 1e-04 to 0.1 | 0.0003473957 | 0.0005712981 | 0.0001785751 | 0.0001261947 | 0.0002425394 |  |  |  |  |  |
| RF | mtry.ratio | 0.0 to 1 | 0.06965079 | 0.06515356 | 0.06720133 | 0.09885725 | 0.1044549 | 0.787 | 0.787 | 0.783 | 0.787 | 0.782 |
|  | replace | NA* | FALSE | FALSE | TRUE | FALSE | TRUE |  |  |  |  |  |
|  | sample.fraction | 0.1 to 1 | 0.3089502 | 0.2036893 | 0.7544046 | 0.9321583 | 0.3614707 |  |  |  |  |  |
|  | num.trees | 1.0 to 2000 | 1077 | 1091 | 1856 | 184 | 1995 |  |  |  |  |  |
| KNN | k | 1 to 50 | 38 | 47 | 32 | 30 | 38 | 0.768 | 0.769 | 0.759 | 0.766 | 0.763 |
|  | distance | 1 to 5 | 2.0585 | 2.216668 | 1.463168 | 1.348855 | 1.437785 |  |  |  |  |  |
|  | kernel | NA* | gaussian | inv | cos | cos | cos |  |  |  |  |  |
| XGBoost | nrounds | 1e+00 to 5000 | 3931 | 4133 | 3378 | 4016 | 4133 | 0.787 | 0.789 | 0.786 | 0.789 | 0.782 |
|  | eta | 1e-04 to 1 | 0.0399705 | 0.04260577 | 0.003673874 | 0.01064548 | 0.0002722646 |  |  |  |  |  |
|  | max_depth | 1e+00 to 20 | 1 | 5 | 15 | 3 | 19 |  |  |  |  |  |
|  | colsample_bytree | 1e-01 to 1 | 0.2924096 | 0.5163034 | 0.4680019 | 0.9048134 | 0.6839875 |  |  |  |  |  |
|  | colsample_bylevel | 1e-01 to 1 | 0.5785542 | 0.8146966 | 0.7952632 | 0.2729495 | 0.7137028 |  |  |  |  |  |
|  | lambda | 1e-03 to 1000 | 25.47094 | 0.01528155 | 91.38381 | 0.05050151 | 0.01227855 |  |  |  |  |  |
|  | alpha | 1e-03 to 1000 | 0.006643897 | 38.24817 | 0.06449964 | 45.28747 | 3.206413 |  |  |  |  |  |
|  | subsample | 1e-01 to 1 | 0.7505099 | 0.3298259 | 0.1600927 | 0.9469936 | 0.7237393 |  |  |  |  |  |

**The replace levels in RF including TRUE, FALSE; The kernel levels in KNN including: rectangular, optimal, epanechnikov, biweight, triweight, cos, logscale*.

| **Learner** | **Parameter set** | **Search space** | **Parameter result** | | | | | **AUC** | | | | |
| --- | --- | --- | --- | --- | --- | --- | --- | --- | --- | --- | --- | --- |
| **CV** | | | **1** | **2** | **3** | **4** | **5** | **1** | **2** | **3** | **4** | **5** |
| ENR | s | 1e-04 to 10000 | 0.004791336 | 0.0002756646 | 0.001668478 | 0.000465869 | 0.00419304 | 0.788 | 0.786 | 0.784 | 0.781 | 0.784 |
|  | alpha | 0e+00 to 1 | 0.6851973 | 0.2804501 | 0.9360801 | 0.6261882 | 0.6326837 |  |  |  |  |  |
| DT | minsplit | 2e+00 to 128.0 | 126 | 10 | 111 | 22 | 2 | 0.760 | 0.769 | 0.768 | 0.754 | 0.762 |
|  | minbucket | 1e+00 to 64.0 | 63 | 31 | 49 | 32 | 39 |  |  |  |  |  |
|  | cp | 1e-04 to 0.1 | 0.0003025149 | 0.0001503902 | 0.0001114313 | 0.0005671278 | 0.0002401699 |  |  |  |  |  |
| RF | mtry.ratio | 0.0 to 1 | 0.0620489 | 0.02784915 | 0.07899587 | 0.09131232 | 0.06062353 | 0.784 | 0.788 | 0.788 | 0.786 | 0.783 |
|  | replace | NA | FALSE | FALSE | FALSE | FALSE | TRUE |  |  |  |  |  |
|  | sample.fraction | 0.1 to 1 | 0.1775025 | 0.5616456 | 0.1562276 | 0.1148639 | 0.1283728 |  |  |  |  |  |
|  | num.trees | 1.0 to 2000 | 813 | 693 | 964 | 1092 | 1056 |  |  |  |  |  |
| KNN | k | 1 to 50 | 30 | 49 | 49 | 45 | 50 | 0.768 | 0.783 | 0.776 | 0.772 | 0.770 |
|  | distance | 1 to 5 | 1.789343 | 3.512196 | 2.726922 | 3.345659 | 3.440494 |  |  |  |  |  |
|  | kernel | NA | cos | rectangular | rank | rectangular | rank |  |  |  |  |  |
| XGBoost | nrounds | 1e+00 to 5000 | 3977 | 4479 | 2942 | 843 | 1545 | 0.791 | 0.790 | 0.788 | 0.785 | 0.784 |
|  | eta | 1e-04 to 1 | 0.009022581 | 0.004956269 | 0.004286549 | 0.1315102 | 0.01517813 |  |  |  |  |  |
|  | max_depth | 1e+00 to 20 | 10 | 19 | 18 | 7 | 19 |  |  |  |  |  |
|  | colsample_bytree | 1e-01 to 1 | 0.2963944 | 0.6166799 | 0.7820289 | 0.6846735 | 0.1843133 |  |  |  |  |  |
|  | colsample_bylevel | 1e-01 to 1 | 0.316349 | 0.3814324 | 0.2790622 | 0.3536997 | 0.4568933 |  |  |  |  |  |
|  | lambda | 1e-03 to 1000 | 2.847048 | 15.3629 | 0.003699077 | 0.001015807 | 0.001004004 |  |  |  |  |  |
|  | alpha | 1e-03 to 1000 | 17.05961 | 1.480238 | 6.317742 | 11.548 | 30.12491 |  |  |  |  |  |
|  | subsample | 1e-01 to 1 | 0.2778729 | 0.1774248 | 0.7404084 | 0.9982821 | 0.6595513 |  |  |  |  |  |

**Table S5.** Hyperparameter Tuning Results for ENR, DT, RF, KNN and XGBoost with 8 Features

**
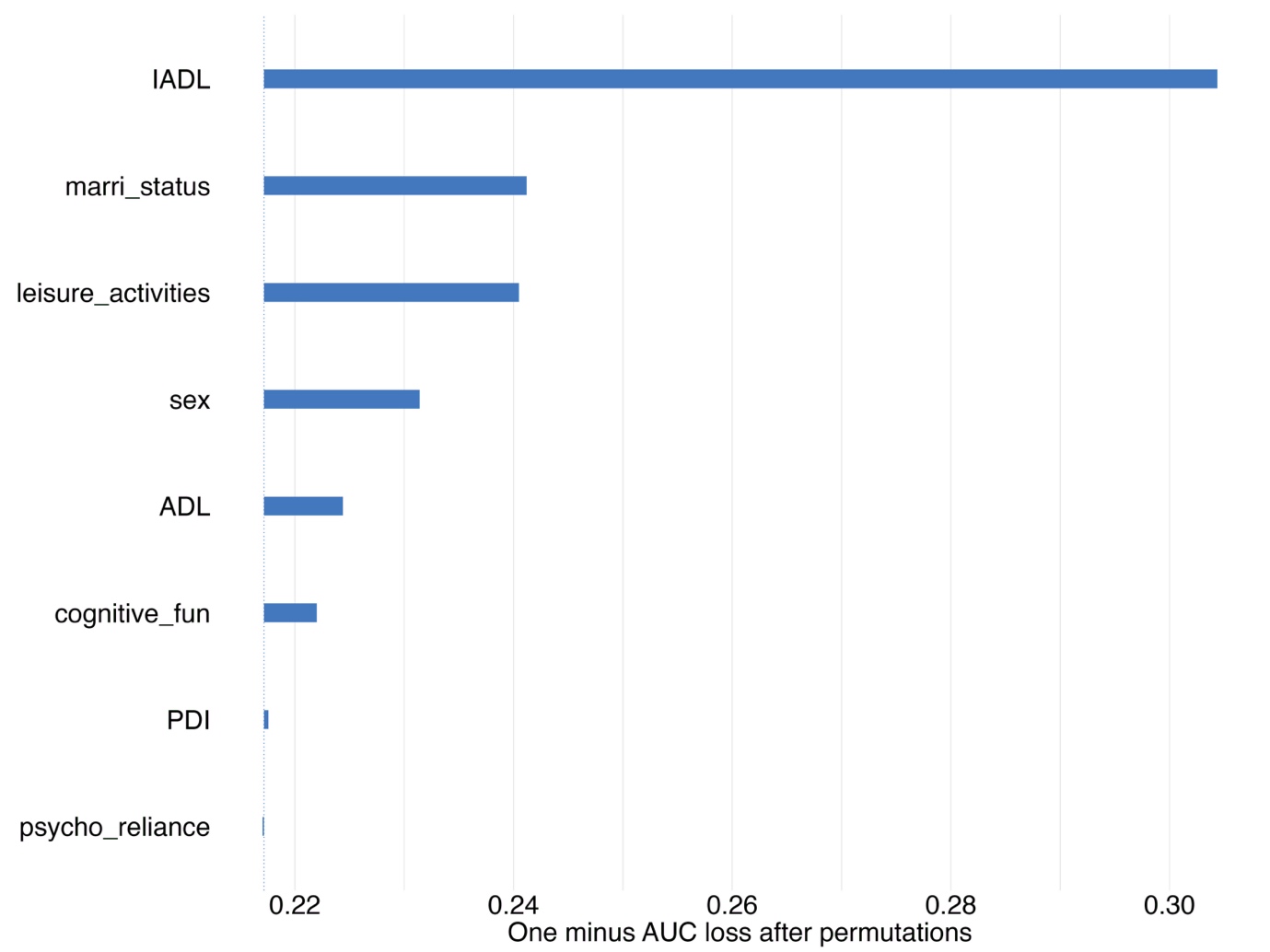
**

**Fig. S1.** Variable Importance of XGBoost-Built Model Predicting 4-Year All-Cause Mortality. in Older Adults with 8 Features. Note. IADL = Instrumental Activities of Daily Living; leisure_activities = leisure activities; marri_status = marital status; ADL = Activities of Daily Living; cognitive_fun = cognitive function; PDI = Plant-Diet Index; psycho_reliance = psychological resilience. For additional explanations regarding the titles, please see Supplementary Material, Table S3. The same below.

**
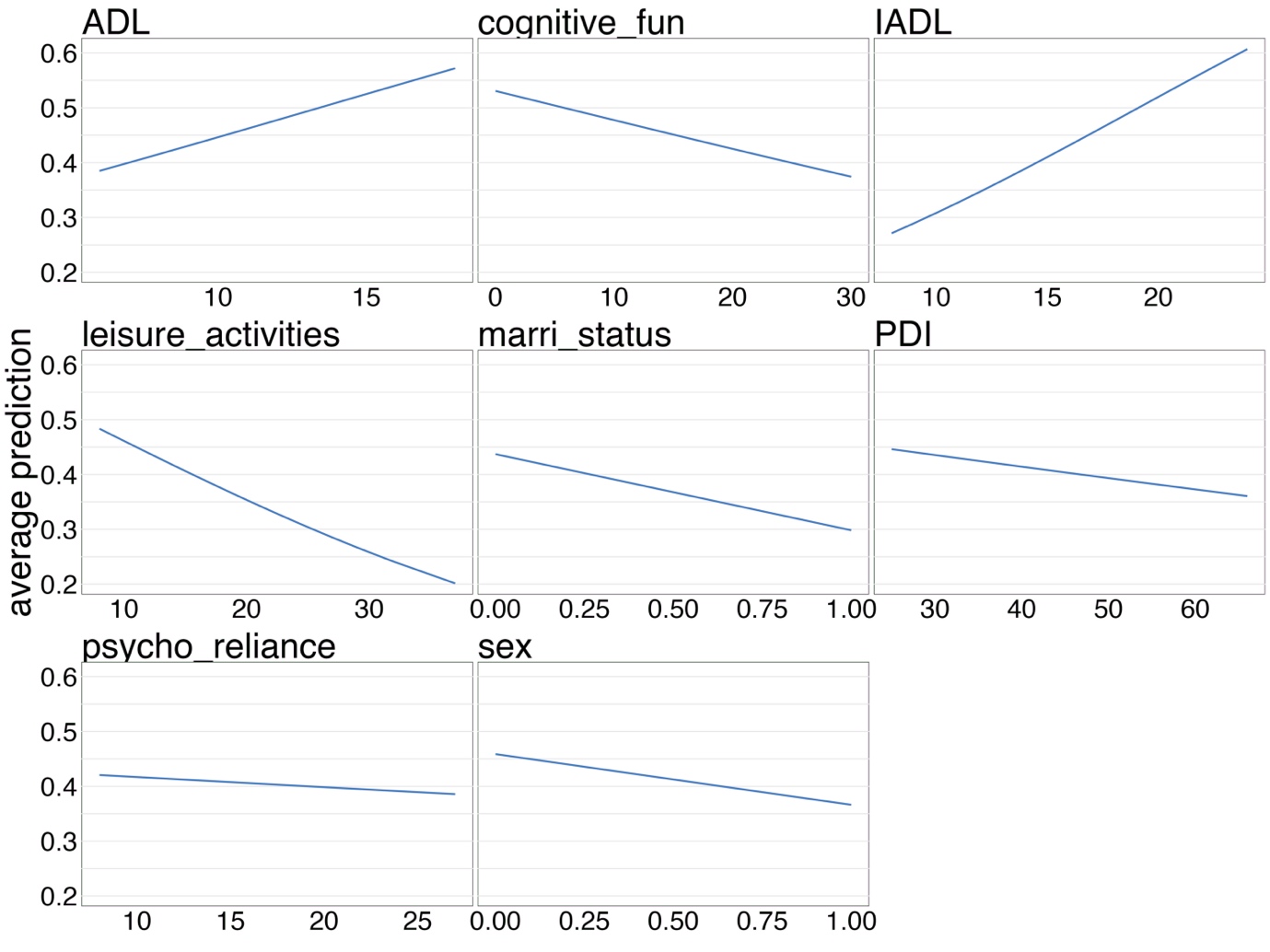
**

**Fig. S2.** Partial Dependence Plots for the XGBoost-Built Model Predicting 4-Year All-Cause Mortality in Older Adults with 8 Features
